# Open Partial Nephrectomy for High-Risk Renal Masses Is Associated with Renal Pseudoaneurysms: Assessment of a Severe Procedure-Related Complication

**DOI:** 10.1155/2015/981251

**Published:** 2015-10-11

**Authors:** M. C. Kriegmair, P. Mandel, N. Rathmann, S. J. Diehl, D. Pfalzgraf, M. Ritter

**Affiliations:** ^1^Department of Urology, University Medical Centre Mannheim, Theodor-Kutzer-Ufer 1-3, 68167 Mannheim, Germany; ^2^Department of Urology, University Medical Centre Hamburg-Eppendorf, Martinistraße 52, 20246 Hamburg, Germany; ^3^Department of Radiology, University Medical Centre Mannheim, Theodor-Kutzer-Ufer 1-3, 68167 Mannheim, Germany

## Abstract

*Objectives.* A symptomatic renal pseudoaneurysm (RPA) is a severe complication after open partial nephrectomy (OPN). The aim of our study was to assess incidence and risk factors for RPA formation. Furthermore, we present our management strategy. *Patients and Methods.* Clinical records of consecutive patients undergoing OPN were assessed for surgical outcome and postoperative complications. Renal masses were risk stratified for tumor complexity according to the PADUA score. Uni- and multivariate analysis for symptomatic RPAs were performed using the *t*-tests and logistic regression. *Results.* We identified 233 patients treated with OPN. Symptomatic RPAs were observed in 13 (5.6%) patients, on average 14 (4–42) days after surgery. Uni- and multivariate analysis identified tumor complexity to be an independent predictor for symptomatic RPAs (*p* = 0.004). There was a significant correlation between RPAs and transfusion and the duration of stay (*p* < 0.001 and *p* = 0.021). Symptomatic RPAs were diagnosed with CT scans and successfully treated with arterial embolization. *Discussion.* Symptomatic RPAs are not uncommon after OPN for high-risk renal masses. A high nephrometry score is a predictor for this severe complication and may enable a risk-stratified followup. RPAs can successfully be located by CT angiography, which enables targeted angiographic treatment.

## 1. Introduction

Over the last decades the incidental diagnosis of small renal masses has increased due to the widespread use of modern imaging modalities such as computed tomography (CT) and magnet resonance imaging (MRI). The detected tumors are generally smaller without causing symptoms such as hematuria and flank pain [1, 2]. Nephron sparing surgery (NSS) has emerged as therapy of choice for renal masses. It offers equivalent oncological outcome and complications with improved postoperative renal function and quality of life compared to radical nephrectomy [3–5].

Whereas the relevance of minimally invasive surgery is rapidly rising, OPN remains the most frequently applied approach, which is especially valuable for difficult renal masses [6–8]. Regardless of the technique, the TRIFECTA criteria defined as safe oncological excision, low ischemia time, and zero complication rate are the ultimate objective [9]. However, especially in terms of complications this goal cannot always be achieved. The two main NSS-related postoperative complications are hemorrhage and urinary leakage [10]. The transfusion rate of PN varies between 4.3% and 20% and in most cases perioperative blood loss can be managed conservatively [6, 10]. In case of an intraparenchymal RPA, however, delayed blood loss can reach a life threatening extent and requires immediate diagnosis and management. A RPA consists of a perivascular collection of blood leaking from an injured vessel. The collection may rupture and cause acute bleeding. The aneurysms may reach extensive size or remain small when early rupture and drainage, for instance, into the collecting system, occurs. According to the literature, symptomatic RPAs after PN occur in up to 5.0% [11–13] and are less frequent in OPN [14]. In general, only symptomatic patients are diagnosed and the actual incidence is difficult to estimate. However, a recent study found asymptomatic RPAs to occur in more than 20% of partial nephrectomies [15]. In most cases symptomatic RPA can successfully be treated with selective arterial embolization [16].

As a high volume center we perform open partial nephrectomy predominantly for high and intermediate risk renal masses. We experienced delayed hemorrhage due to an intraparenchymal RPA to be the most frequent and most dangerous severe complication. The aim of our study was to assess the incidence and risk factors for false aneurysm and to present our management and outcome.

## 2. Patients and Methods

### 2.1. Study Design and Data Collection

The study was approved by the local ethical committee (2013-830-MA). We analyzed consecutive partial nephrectomies of seven different surgeons between 2013 and 2015. Surgeon's experience was divided into three levels: beginners (less than 50 OPN), advanced (50–200 OPNS), and experts (more than 200 OPN). Medical charts were assessed for patient characteristics such as age, BMI, and ASA score and tumor characteristics such as tumor size, nephrometry using the PADUA score, and histopathological findings [17]. Surgical data comprises operation time (OT), estimated blood loss (EBL), ischemia time (IT), and opening of the collecting system (CS). Postoperative complications were assessed using the Clavien classification with a followup of 30 days [18]. In addition, patients with a postoperative RPA were further assessed for complication management in terms of arterial embolization.

### 2.2. Surgical Technique

All patients underwent combined anesthesia including the placement of a peridural catheter, unless contraindicated. OPN was performed through a retroperitoneal approach with a 10–15 cm flank-incision above the 11th rib, gaining access to the retroperitoneal cavity. Complete exposure of the organ and the renal hilum allowed for identification and marking of the renal vessels and the ureter with vessel loops. Resection of the tumor was either performed with clamping of the kidney vessels or in zero ischemia technique as described before [6]. Afterwards, bleeding vessels are tied with polyfilament sutures. In case of need, urinary CS defects are repaired using monofilament sutures. Resection edges are adapted by secure renorrhaphy using one or two layers of monofilament sutures. On demand surgeons applied a hemostatic patch (TachoSil) on the resection surface prior to renorrhaphy. An oncologically safe resection was proven by intraoperative frozen section. In case of a drain insertion the tube was placed close to the resection side and channeled at the lower medial pole of the flank incision. The drain was fixed with a monofilament suture and no suction was applied. Finally, the kidney was again covered by the perirenal fat and the wound was closed in layers. A ureter stent or a nephrostomy tube was not inserted.

### 2.3. Diagnosis and Management of Pseudoaneurysms

Clinical symptoms that arouse suspicion of delayed hemorrhage and a possible RPA after OPN included persistent gross hematuria, significant drop in hemoglobin level (>2 mg/dL/24 h), and sudden and severe flank pain.

In the respective cases we performed a multiple phase contrast enhanced (CE) computed tomography (CT) including a native CT scan. For identification of possible arterial bleeding, arteriovenous fistula or RPA CT angiography (CTA) was performed with injection of 105 mL contrast media (Imeron 400 MCT 400 mg iodine/mL; Bracco Imaging Germany) using a peripheral venous access with a flow rate of 4 mL/s, followed by a saline injection of 40 mL with identical flow rate. Using bolus tracking and a region of interest, which was placed in the abdominal aorta at the level of the diaphragm, the arterial phase CT scan was initiated at a threshold of 100 Hounsfield units (HU) and an additional delay of 8 s. The arterial phase CT scan was followed by a portal-venous phase scan 55 s after arterial phase imaging. The CT scans were performed in breath hold in a deep-inspiration state. If the patient was not able to follow the breath hold instruction (e.g., because of reduced general state), a shallow breathing was tolerated. Dose modulation options were used (CARE Dose 4D; Siemens Medical Solutions). Multiplanar data reconstruction was performed to identify renal artery anatomy and possible feeding vessels of the RPA for planning of the transarterial catheter intervention. If the GFR was impaired the recommendations of the ESUR are followed and the patient was hydrated with an intravenous infusion of saline before and after CE CT.

In case of positive identification of a RPA the patient was directly transferred to the angiography suite. Angiography was performed using a transarterial femoral access with a 5-F sheath. A standard 4-F diagnostic catheter was advanced in the renal artery if possible without additional administration of contrast media with the knowledge given from CTA. Selective renal angiography was performed. If the RPA or feeding vessel could not be identified in posterior-anterior projection additional left open and right open angle projection (±30° to 45°) angiography was performed. The feeding vessel was probed with a 3-F coaxial microcatheter system (Progreat; Terumo, Eschborn, Germany) in a superselective approach. The tip of the microcatheter was placed as close as possible to the RPA to only embolize the RPA and spare as much renal parenchyma as possible. Pushable microcoils were used for embolization. If coil-dislocation was feared, detachable hydrocoils (Azur peripheral hydrocoil, Terumo Medical Corporation) were used. The microcoils were either hydrogel covered (Azur peripheral hydrocoil, Terumo Medical Corporation) or fiber covered platin coils (Boston Scientific). The coil size was determined by the approximately vessel size and experience of the interventionalist. Initially two microcoils were placed and after 5 min control of embolization was performed with injection of 1 mL contrast media into the microcatheter using a 1 mL syringe. If rest perfusion of the RPA was still visible one or two more microcoils were introduced depending on the volume of rest perfusion and the control was performed again after 5 min. This was repeated until total occlusion of the RPA.

In case of a life-threatening state and hemodynamic instability of the patient, the patient was transferred directly to the angiography suite and transarterial angiography was performed as described above without prior CTA. If superselective catheterization was impossible due to elongated arteries the segment artery, the main branch artery, or the renal artery itself was occluded in case of hemodynamic instability.

### 2.4. Statistical Analysis

To check for differences in our respective subsamples, two-group mean-comparison *t*-tests, Wilcoxon-Mann-Whitney test, and Pearson's chi-squared tests were performed.

To evaluate the association between potential risk factors and the occurrence of postoperative RPA, uni- and multivariate logistic regressions were run. Statistical software STATA was used (version 14 for Windows, StataCorp LP, College Station, TX).

## 3. Results

We assessed data of 240 patients admitted for open partial nephrectomy by seven different surgeons at our university medical center. In seven patients intraoperative conversion to radical nephrectomy was performed due to vascular tumor invasion or an insufficiently perfused remaining kidney, leaving 233 cases for final analysis.

Table 1 illustrates the patient and tumor characteristic of the study population. Median age and BMI were 63 (73–55) years and 25.6 (29.7–24.1) kg/m^2^, respectively. The median ASA score was 2 (3-2) and approximately two out of three patients (67.7%, *n* = 158) were male. Median tumor size was 3.0 (4.2–2.2) cm and 76.4% (*n* = 178) of the tumors were malignant, of which the majority (67.2%, *n* = 120) were classified as clear cell carcinomas. Nephrometry revealed a PADUA score of 8-9 (intermediate risk) for 40.5% (*n* = 94) and ≥10 (high risk) for 30.4% (*n* = 71) of the tumors.

We identified 16 patients (6.8%), who presented with a delayed hemorrhage. In 13 cases a symptomatic RPA was diagnosed. The remaining 3 patients suffered from a ureteric stone and a urine extravasation, respectively, and for one patient on oral anticoagulation, no pathology could be identified. Patients with a symptomatic RPA accounted for more than 50% of all severe complications. As shown in Table 2 there was no significant difference in OT and EBL between the patients without and with a RPA. Overall, median OT was 2.5 (3.1–2.0) h and median EBL 200 (300–100) mL. For the majority of patients warm ischemia was required and there was no significant difference between the patients with and without RPA: 84.6% (*n* = 11/13) versus 75.2% (*n* = 165/220). A similar situation was found in terms of length of ischemia, which was 20.2 ± 10.0 min for all patients and with 20.8 ± 10.1 min slightly longer in the RPA group. During the resection of the tumor the CS was opened in 92.1% (*n* = 12/13) of the cases in the group of patients with a consecutive symptomatic RPA; this is significantly more often than in patients without a RPA (65.9, *n* = 145/220) (*p* = 0.048). According to this finding, the PADUA score was significantly different between the two groups (*p* < 0.001). The median PADUA score was 8 (11–7) in patients without RPA and 10 (11-10) in those presenting a delayed hemorrhage due to a RPA. Tumor size was only slightly higher (+0.48 cm) in the RPA group and the difference was not significant (*p* = 0.239). Patients with a symptomatic RPA required blood transfusion significantly more often during the initial or hospital stay or after readmission (*p* < 0.001). Almost two out of three patients (61.5%, *n* = 7/13) with delayed hemorrhage were transfused, whereas only 8.6% (*n* = 19/220) of patients without a RPA needed blood transfusion. Corresponding patients with a RPA had a significantly longer hospital stay (*p* = 0.007). With regard to the surgeons' experience, there was no significant difference between the two groups, although in the RPA group proportionally more beginners (30.8% versus 22.4%) and fewer experts (38.5% versus 47.2%) performed surgery.

The results of the univariate logistic regression for the occurrence of a symptomatic RPA are given in Table 3. The PADUA score was found to be a predictor for the formation of symptomatic RPA after OPN (OR = 1.959, CI = 1.294–2.965, *p* = 0.001). Opening of the CS was not found to be significant in logistic regression, although there was a strong trend towards significance (OR = 6.213, CI = 0.782–48.719, *p* = 0.082). Furthermore, there was a significant correlation between a symptomatic RPA and transfusion rate as well as duration of stay (OR = 16.337, CI = 4.859–54.929 *p* < 0.001 and OR = 1.108, CI = 1.016–1.209, *p* = 0.021). No significant correlation was found for the other variables including experience of the surgeon, tumor size, and the absence of hemostatic agents. We additionally performed a multivariate analysis with all significant variables of the univariate analysis, showing PADUA as independent predictor (OR = 2.197, CI = 1.294–3.731, *p* = 0.004). Furthermore, transfusion rate was significant in the multivariate analysis (OR = 23.648, CI = 4.084–136.91).

The 13 patients presenting a delayed hemorrhage became symptomatic on the 14th day after surgery in average, with a range from 4 to 42 days. The majority of patients suffered from gross hematuria (76.9%, 10/13), eventually leading to the formation of a vesical tamponade, which was observed in 3/13 cases (30.8%). One tamponade needed endoscopic evacuation under general anesthesia. Bleeding caused a significant drop in hemoglobin in 76.9% (10/13) of the cases. Consequently, 8/13 patients (61.5%) became symptomatic and required blood transfusion. Only 3/13 (30.8%) patients suffered from flank pain.

**Figure 1:**
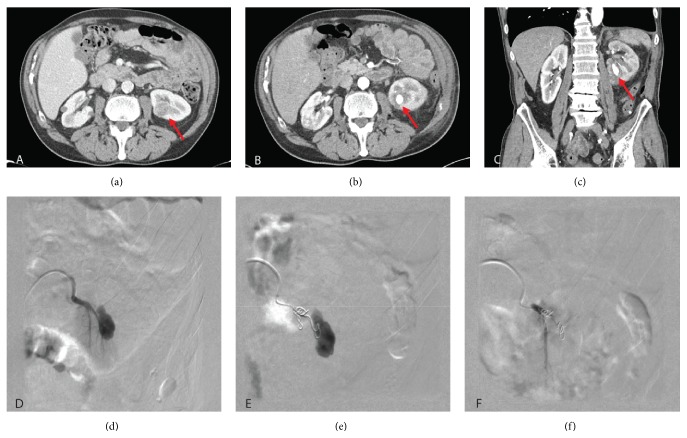
Preoperative CT scan, CTA, and arterial embolization of PRA. (a) Preoperative CT-scan of a central endophytic renal mass (red arrow). ((b), (c)) Transversal and coronary view of a postoperative CT-scan showing a RPA in the former resection area (red arrow). (d) Digital subtraction angiography (DSA) of the lower pole segmental renal artery showing the RPA. (e) Superselective DSA via microcatheter after embolization with 4 microcoils showing remaining perfusion of the RPA. (f) Superselective DSA via microcatheter after another 4 min: total occlusion of the RP with rest perfusion of the lower pole.

In general, diagnosis was confirmed with CT-angiography as exemplified in Figure 1. However, for one patient (1/13) we performed a MRI-scan due to a severe renal failure in the setting of a single kidney. Additionally, the respective patient was treated conservatively with blood transfusion and thankfully gross hematuria ceased after 48 hours. For all other patients (12/13) arterial embolization as illustrated in Figure 1 was successful and patients were asymptomatic after the procedure.

## 4. Discussion

Partial nephrectomy is the therapy of choice for renal masses and should be performed whenever technically feasible. Due to increased incidental diagnosis of kidney tumors it gains further importance [3]. Independent of the surgical approach, PN harbors two major procedure-related renal complications, namely, urinary leakage and hemorrhage [10]. Bleeding may occur during surgery or the early postoperative period often manageable by blood transfusion. Depending on risk factors like tumor size, surgical approach or parameters such as IT the rate of blood transfusion vary between 4% and 20% [6]. Delayed hemorrhage may be the result of the formation and subsequent rupture of a RPA, usually becoming symptomatic 10–14 days after surgery [14]. Symptoms are flank pain or gross hematuria that can lead to life-threatening blood loss. Singh and Gill proposed two different mechanisms that lead to the formation of RPAs after partial nephrectomy. Firstly, perforce vessel injury during the resection of the tumor leads to blood leakage into the surrounding tissue eventually communicating with the intravascular space. Secondly, sutures are incidentally placed through a vessel that may cause bleeding into the parenchyma that is not noticed during the operation [19]. Controlling mechanisms like decreased blood flow, tamponading effects, and blood coagulation may fail in the postoperative time when the patient is mobilized and blood pressure increases.

In general, only symptomatic RPAs are diagnosed, since their finding requires advanced imaging modalities such as contrast enhanced CT scan or angiography [11]. A systematic review of 5229 patients found symptomatic RPAs in 1% and 1.98% of OPNs and minimally invasive PNs, respectively [14]. Other studies, however, claimed the incidence of bleeding RPAs to be up to 5%, whereas the laparoscopic approach was generally accompanied by more symptomatic RPAs [11, 20]. A recent study assessed the incidence of asymptomatic RPA by postoperative CT scans and found 18.2% of the patients after laparoscopic PN presenting a RPA. The incidence after open partial nephrectomy was 12.3% [21]. One explanation for the higher RPA rate after laparoscopic PN is, according to the two mechanisms proposed by Singh and Gill, the less accurate suturing and the employment of larger needles during the minimally invasive approach [19]. In our series we identified 13 (5.8%) symptomatic RPAs, which is remarkably higher than what was found in previous studies for OPN. As a high volume center we generally perform OPN for challenging and larger renal masses and partly treat small and low-risk tumors with minimally invasive approaches. This assortment may eventually lead to a risk selected study population. Furthermore, comparable studies assessed data a few years ago when the attempt of performing NSS was not demanded for any renal mass [14]. Indeed, 70.9% (165) of the 233 cases assessed presented a high or intermediate-risk nephrometry score (PADUA > 8). Our data suggest that the previous assumption that delayed hemorrhage due to a RPA is a problem mainly associated with minimally invasive PN should be put into perspective [14, 20]. OPN harbors a significant risk of RPA formation as well, when high-risk renal masses are excised. Gill et al. proposed that especially the resection of endophytic and central tumors harbors the risk of vessel injury, either direct or by suturing, and consequent RPA formation, which supports the assumption [19, 22]. Corresponding, Nadu et al. presented a series of 53 centrally located tumors, in which the incidence of RPA was 7.5% [23].

A recent study identified involvement of the renal sinus to be an independent risk factor for the formation of asymptomatic RPA [15]. Our analysis identified tumor complexity measured by the PADUA score to be an independent risk factor for the occurrence of RPAs after partial nephrectomy [17]. Indeed the average PADUA score among the 13 patients presenting a symptomatic RPA was 10.1 ± 1.2 with 8 high-risk tumors and only one low-risk renal mass. Nephrometry helps to identify high-risk patients for the formation of RPA. These patients might benefit from a risk-adapted followup including frequent clinical checkups. Respective patients should also be informed on their increased risk and made aware for symptoms of a RPA. Avoidance of high blood pressure and prolonged postoperative physical rest may also be preventive. Opening of the CS was significantly different between the two groups and showed a strong trend towards significance in the univariate logistic regression. Tumor size or malignancy was not associated with a higher rate of RPAs, which goes along with previous studies that proved tumor size alone to be insufficient for the prediction of complications [10]. Whereas our analysis proved adverse tumor anatomy to be associated with a higher likelihood of RPAs, patients' characteristics like BMI or age did not show any coherence, although BMI was found to be associated with an increased overall complication rate of partial nephrectomy before [17]. In addition assessment of surgical experience could not detect differences between the distinct groups of surgeons. In our series a less experienced surgeon did not eventually lead to a higher rate of symptomatic RPAs. Furthermore, no coherence between the absences of hemostatic agents used in combination with renorrhaphy and RPA formation was observed. This is in accordance with previous studies that failed to show an advantage of coagulant patches in terms of complications and functional outcome [24]. Summarizing the only identifiable risk factor so far remains the tumor complexity assessed with nephrometry scores, in particular the PADUA score. In general, a higher score implicates a more centrally located and endophytic renal mass. Our findings seem to be reasonable, regarding the pathophysiology of RPAs proposed by Singh and Gill, which basically holds inevitable vessel injury accountable for the formation of RPAs. In case of a central mass also an experienced surgeon has to necessarily cut through larger segmental arteries.

Surprisingly, ischemia time or estimated blood loss, which serve as surrogate parameter for tumor complexity, was not associated with the formation of symptomatic RPAs. Transfusion rate and length of hospital stay were found to significantly correlate with the occurrence of a PRA. This coherence, however, is surely due to the fact that blood transfusion and a longer stay are a consequence of a symptomatic RPA.

Beside risk factors for RPA, we assessed the time of occurrence of RPAs and their clinical symptoms. In this series patients were symptomatic 14 (4–42) days after OPN. Other studies confirmed that RPAs become symptomatic on average 10–14 days after partial nephrectomy, although there is a large variance [11, 14]. Four patients with a RPA presented symptoms as recently as 20 days or later after surgery. This necessitates not only a close followup of risk-associated patients (e.g., a high PADUA score) but also sufficient information of the patient concerning possible delayed symptoms. The majority of patients suffered from gross hematuria, which is consistent with other studies [11, 25]. Flank pain due to perirenal hematoma may also be a sign of a ruptured RPA but is less frequent. However, symptoms of delayed hemorrhage need rapid investigation as it is a potentially life threatening situation [21, 26]. In our series almost 80% of the patients presented a significant drop in hemoglobin and more than 60% required blood transfusion.

We used CT-angiography for fast diagnosis in the case of delayed hemorrhage, which has been proven to be reliable and valid in detecting RPA aneurysms and other potential causes [15, 27]. A ruptured RPA is the most common reason for delayed bleeding after PN. For that reason other groups claimed to perform angiography first to be able to directly carry out arterial embolization [11, 23]. We performed CT-angiography first when signs of delayed hemorrhage occurred, since it rules out other reasons of bleeding, not detectable in angiography, and consequently helps to spare the more invasive approach (in our group 3/16 patients). Furthermore, the localization of the RPA can be easily identified in the CT-angiography. Together with the imaging of the arterial supply of the kidney, this facilitates a more targeted arterial embolization, eventually sparing time and radiation doses [27]. Furthermore, it might help to reduce contrast agent doses and hence impairment of renal function, which is important especially after partial nephrectomy. The approach of first performing a CT-angiography and, if required, an arterial embolization in the emergency setting requires excellent logistic and short distances [27]. With this management strategy we were able to identify all 13 cases with asymptomatic RPA from a total of 16 patients presenting with signs of delayed hemorrhage. Whereas 1 case was treated conservatively, the remaining 12 patients with symptomatic RPA were successfully treated with arterial embolization.

There are several limitations to our study. First and foremost are the limitations inherent to retrospective analyses. Furthermore, the study only assesses OPN. Data on minimally invasive PN is missing. To sufficiently compare incidence and risk factors of PRA after OPN and minimally invasive PN as well as to evaluate the surgical approach as a risk factor, further multicentre centre studies are required. The population furthermore is risk selected and contains more anatomically complex renal masses. Finally, information on relevant comorbidities was missing. These covariates may have influenced analysed outcome variables.

Despite these limitations, this study adds important knowledge to the literature and is the first study to identify independent predictors for the formation of symptomatic RPA after OPN. We also show that the low incidence of RPA after OPN demonstrated in previous studies might be underestimated in an era when the indication of nephron sparing surgery is extended to very complex tumours.

## 5. Conclusion

Delayed hemorrhage due to a RPA is one of the most frequent severe complications after OPN. The majority of patients present with gross hematuria and a significant drop in hemoglobin, often necessitating blood transfusion. A high nephrometry score (PADUA) is an independent predictor for the occurrence of a symptomatic RPA. CT angiography allows for a safe diagnosis, taking into account possible differential diagnosis, and may help to facilitate a rapid and uneventful angiographic intervention.

## Figures and Tables

**Table 1:** Patient characteristics of the 233 open partial nephrectomies.

Age in years, median (IQR)	63 (73–55)
BMI in kg/m^2^, median (IQR)	25.6 (29.7–24.1)
ASA, median (IQR)	2 (3-2)
Male, % (*n*)	67.4 (157)
Tumor side, % (*n*)	
Right	49.1 (114)
Left	50.9 (119)
Tumor size in cm, median (IQR)	3.0 (4.2–2.2)
Malignant, % (*n*)	76.4 (178)
Histology if malignant tumour, % (*n*)	
Papillary type	21.5 (38)
Clear cell	67.2 (120)
Chromophobe	7.9 (14)
Others	3.4 (6)
PADUDA	
7-8, % (*n*)	29.1 (68)
8-9, % (*n*)	40.5 (94)
≥10, % (*n*)	30.4 (71)

**Table 2:** Surgical outcome.

	All patients	Patients without aneurysm	Patients with aneurysm	*p* value
Patients	233	220	13	
OT in h, median (IQR)	2.5 (3.1–2.0)	2.5 (3.1–2.0)	2.5 (3.3–2.0)	0.802
EBL in mL, median (IQR)	200 (300–100)	200 (300–100)	200 (400–100)	0.343
Ischemia, % (*n*)	76.4 (178)	75.2 (165)	84.6 (11)	0.443
Ischemia time (min), mean ± SD	20.2 ± 10.0	20.1 ± 10.0	20.8 ± 10.1	0.846
Hemostatic agent, % (*n*)	51.1 (119)	52.6 (116)	38.5 (5)	0.323
Opening of the collecting system, % (*n*)	67.4 (157)	65.9 (145)	92.1 (12)	**0.048**
Transfusion, % (*n*)	11.6 (27)	8.9 (20)	61.5 (8)	<**0.001**
Duration of stay (days), median (IQR)	6 (8–6)	6 (8–6)	10 (12–7)	**0.007**
PADUA, median (IQR)	8 (11–7)	8 (10–7)	10 (11-10)	<**0.001**
Tumor size in cm, median (IQR)	3.0 (4.2–2.2)	3.0 (4.0–2.2)	3.9 (4.5–2.6)	0.234
Expertise of surgeon, % (*n*)				
Beginner	22.8 (53)	22.4 (50)	30.8 (4)	0.751
Advanced	30.0 (70)	30.4 (67)	30.8 (4)	
Expert	47.2 (110)	47.2 (104)	38.5 (5)	

**Table 3:** Univariate logistic regression for occurrence of RPA.

	OR	CI	*p*
Age	0.985	0.946–1.027	0.482
BMI	1.027	0.915–1.151	0.654
Male	1.094	0.326–3.675	0.885
Malignant	1.765	0.380–8.224	0.469
Tumor size	1.148	0.879–1.497	0.312
PADUA	1.959	1.294–2.965	**0.001**
Blood loss	1.000	0.998–1.002	0.827
Ischemia	1.811	0.389–8.431	0.449
Opening of the CS	6.213	0.792–48.719	0.082
Advanced	0.738	0.176–3.101	0.679
Experts	0.594	0.153–2.312	0.453
Transfusion	16.337	4.859–54.929	<**0.001**
Duration of stay	1.108	1.016–1.209	**0.021**
